# Immunological classification of gliomas based on immunogenomic profiling

**DOI:** 10.1186/s12974-020-02030-w

**Published:** 2020-11-27

**Authors:** Qiushi Feng, Lin Li, Mengyuan Li, Xiaosheng Wang

**Affiliations:** 1 Biomedical Informatics Research Lab, School of Basic Medicine and Clinical Pharmacy, China Pharmaceutical University, Nanjing, 211198 China; 2 Cancer Genomics Research Center, School of Basic Medicine and Clinical Pharmacy, China Pharmaceutical University, Nanjing, 211198 China; 3 Big Data Research Institute, China Pharmaceutical University, Nanjing, 211198 China

**Keywords:** Glioma, Lower-grade glioma, Glioblastoma, Tumor immune microenvironment, Immunological classification, Immunogenomic profiling, Clustering

## Abstract

**Background:**

Gliomas are heterogeneous in the tumor immune microenvironment (TIM). However, a classification of gliomas based on immunogenomic profiling remains lacking.

**Methods:**

We hierarchically clustered gliomas based on the enrichment levels of 28 immune cells in the TIM in five datasets and obtained three clusters: immunity-high, immunity-medium, and immunity-low.

**Results:**

Glioblastomas were mainly distributed in immunity-high and immunity-medium, while lower-grade gliomas were distributed in all the three subtypes and predominated in immunity-low. Immunity-low displayed a better survival than other subtypes, indicating a negative correlation between immune infiltration and survival prognosis in gliomas. *IDH* mutations had a negative correlation with glioma immunity. Immunity-high had higher tumor stemness and epithelial-mesenchymal transition scores and included more high-grade tumors than immunity-low, suggesting that elevated immunity is associated with tumor progression in gliomas. Immunity-high had higher tumor mutation burden and more frequent somatic copy number alterations, suggesting a positive association between tumor immunity and genomic instability in gliomas.

**Conclusions:**

The identification of immune-specific glioma subtypes has potential clinical implications for the immunotherapy of gliomas.

**Supplementary Information:**

The online version contains supplementary material available at 10.1186/s12974-020-02030-w.

## Background

Gliomas comprise nearly 80% of all brain malignancies and included lower-grade glioma (LGG) and glioblastoma (GBM) [1]. LGG has lower grades (II, III) and a more favorable prognosis, while GBM has the highest grade (IV) and a more unfavorable prognosis [1]. Both LGG and GBM are heterogeneous in molecular profiles. For example, the TCGA network classified LGG into three subtypes: *IDH* mutation and 1p/19q codeletion, *IDH* mutation and no 1p/19q codeletion, and *IDH* wild-type [2]. Based on gene expression profiles, Verhaak et al. identified four molecular subtypes of GBM: proneural, neural, classical, and mesenchymal [3]. Certain studies have investigated the immunological heterogeneity of gliomas based on tumor immune signatures [4**–**5]. Doucette et al. found that antitumor immune responses predominated in the mesenchymal subtype of GBM [4]. Wu et al. identified immune-specific subtypes in diffuse LGG [5]. A recent study [6] comprehensively analyzed the tumor microenvironment of the brain and demonstrated multifaceted enrichment of immune cells within gliomas and brain metastasis.

Recently, cancer immunotherapies, such as immune checkpoint blockade [7] and chimeric antigen receptor T cell immunotherapy [8], have been utilized in treating various malignancies [9]. Nevertheless, currently, these strategies are beneficial to only a subset of cancer patients. Thus, identifying the factors determining the different immunotherapeutic responses is crucial for improving the cancer immunotherapeutic responsiveness. Some such factors have been identified, such as PD-L1 expression [10], DNA mismatch repair deficiency [11], and tumor mutation burden (TMB) [12]. Also, the “hot” tumors having dense T cell infiltration are more likely to respond to immunotherapy [13]. Thus, differentiating “hot” tumors from “cold” tumors may facilitate the selection of cancer patients responsive to immunotherapy. In a previous study [14], we developed a method to identify three immune-specific subtypes of triple-negative breast cancers based on immunogenomic profiling, which had high, medium, and low levels of immune infiltration, respectively. Using similar methods, other investigators have identified immune-specific subtypes in other cancer types, such as lung cancer [15] and LGG [5].

In this study, we performed clustering analyses of gliomas based on the enrichment levels of 28 immune cells in the tumor microenvironment in five different datasets. We identified three subtypes of gliomas: immunity-high, immunity-medium, and immunity-low. We compared molecular and clinical features between these subtypes, including pathways, gene ontology, genomic features, tumor progression, and clinical outcomes. The identification of immune-specific subtypes may provide new insights into the pathogenesis of gliomas and potential clinical implications for the immunotherapy of this cancer.

## **Methods**

### Datasets

We downloaded five gene expression profiling datasets for gliomas, including TCGA-glioma (GBM and LGG) from the TCGA data portal (https://portal.gdc.cancer.gov/), GSE16011 [16] from the NCBI gene expression omnibus (https://www.ncbi.nlm.nih.gov/geo/), and CGGA301, CGGA325, and CGGA693 from the Chinese Glioma Genome Atlas (http://www.cgga.org.cn/). We also downloaded the somatic mutation and somatic copy number alteration (SCNA) profiling datasets for TCGA-glioma from the genomic data commons data portal (https://portal.gdc.cancer.gov/). A summary of these datasets is presented in Additional file 1: Table S1.

### Single-sample gene set enrichment analysis

We used the single-sample gene set enrichment analysis (ssGSEA) score [17] to quantify the enrichment level of an immune cell or signature, pathway, or biological process in a tumor sample. Based on gene expression profiles, ssGSEA calculates the enrichment score of a gene set in a sample, which represents the degree to which the genes in the gene set are coordinately up- or downregulated in the sample. The gene sets representing immune cells or signatures, pathways, and biological processes were included in the analysis (Additional file 2: Table S2).

### Clustering analysis

We hierarchically clustered gliomas based on the enrichment levels of 28 immune cell types. The 28 immune cell types included CD56-bright natural killer (NK) cells, effector memory CD4 T cells, eosinophil, CD56-dim NK cells, type 17 T helper cells, activated B cells, monocytes, memory B cells, activated CD4 T cells, type 2 T helper cells, plasmacytoid dendritic cells, neutrophils, macrophages, effector memory CD8 T cells, myeloid-derived suppressor cell (MDSC), immature B cells, T follicular helper cells, NK cells, immature dendritic cells, mast cells, type 1 T helper cells, activated dendritic cells, central memory CD4 T cells, gamma delta T cells, central memory CD8 T cells, regulatory T cells, activated CD8 T cells, and natural killer T cells [18].

### Calculation of immune score and tumor purity

We used ESTIMATE [19] to calculate the immune score and tumor purity for each tumor sample. ESTIMATE evaluates immune scores (the fraction of immune cells) in tumor samples based on immune gene expression signatures. The immune score represents the immune infiltration level in the tumor.

### Calculation of gene amplification frequencies

We calculated the amplification frequency of a gene in a group of tumor samples as the proportion of the tumor samples with the copy number gain in the gene based on the SCNA profiling dataset for TCGA-glioma.

### Survival analysis

We compared the overall survival (OS) and disease-free survival (DFS) between the immune-specific subtypes of gliomas. We used Kaplan–Meier curves to show the survival time differences and the log-rank test to evaluate the significance of survival time differences.

### Pathway and gene ontology analysis

We identified the KEGG [20] pathways highly enriched in immunity-high and immunity-low gliomas by GSEA [21] with a threshold of adjusted *P* value < 0.05. We identified the gene modules (gene ontology) highly enriched in immunity-high and immunity-low gliomas using WGCNA [22].

### Quantification of molecular and genomic features

The TMB of a tumor sample was the total count of its somatic mutations. We used the MATH algorithm [23], which measures the width of the allele frequency distribution, to evaluate the intratumor heterogeneity (ITH) scores of tumor samples. The ITH scores were calculated using the function “math.score” [23] in R package “maftools” with the input of “maf” files. We used GISTIC2 [24] with the input of “SNP6” files to calculate arm-level SCNA frequencies and focal SCNA levels for each tumor sample and compared them between immunity-high and immunity-low gliomas. The “maf” and “SNP6” files were downloaded from the TCGA data portal (https://gdc-portal.nci.nih.gov/).

### Class prediction

We first normalized attribute values (immune cell enrichment levels or ssGSEA scores) by *Z* score and transformed all attribute values into the range between -3 and 3 by setting an attribute value as 3 if it was greater than 3 and setting an attribute value as -3 if it was less than -3. We utilized the Random Forest (RF) classifier [25] to predict the glioma subtypes. In the RF, the number of trees was set to 500, and the features included all 28 immune cells. We evaluated the classification performance using the accuracy and the weighted F-score. We performed the class prediction using the R package “randomForest”.

### Statistical analysis

We used Spearman’s correlation test to evaluate the correlation between pathway activities (ssGSEA scores) and immune scores calculated by ESTIMATE [19] in gliomas. We compared the enrichment levels of stemness and epithelial-mesenchymal transition (EMT) scores between immunity-high and immunity-low gliomas using the Mann–Whitney *U* test. In comparing the frequencies of gene somatic mutations and SCNAs between immunity-high and immunity-low gliomas, we used Fisher’s exact test. We compared the ratios of two different immune signatures between immunity-high and immunity-low gliomas using Student’s *t* test. The ratios were the base-2 log-transformed values of the mean expression levels of all marker genes in the first immune signature divided by those of all marker genes in the second immune signature. We used the Benjamini-Hochberg method [26] to adjust for multiple tests.

## Results

### Identifying glioma immune subtypes based on immunogenomic profiling

**Fig. 1:**
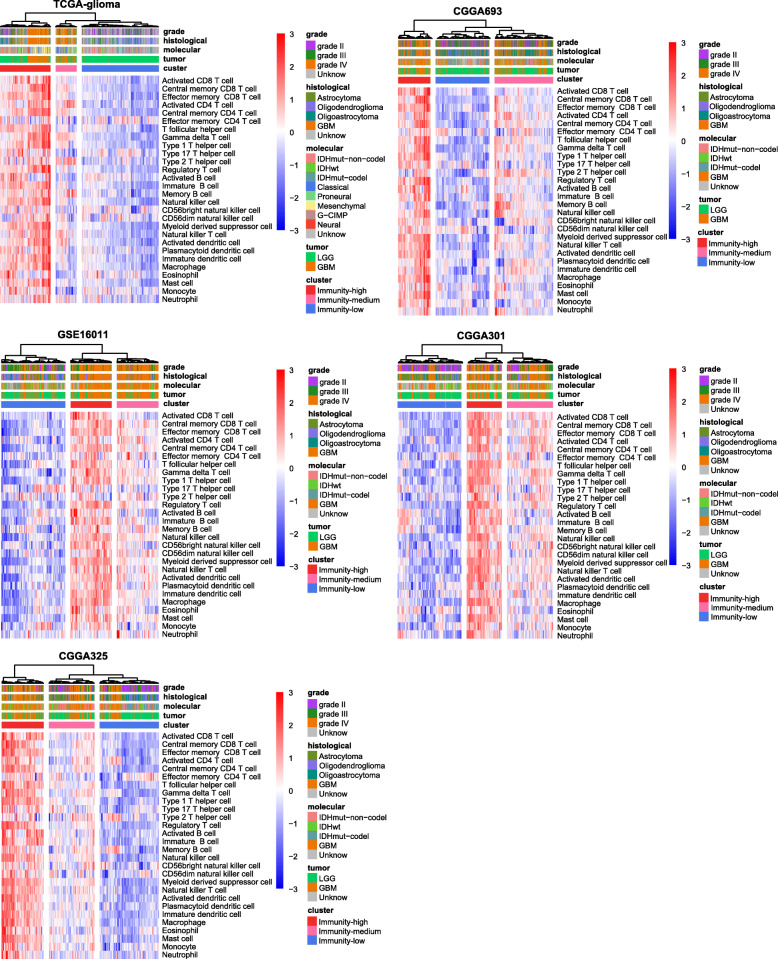
Hierarchical clustering of gliomas based on the enrichment levels of 28 immune cell types in five different datasets. Three clear clusters in all five datasets: immunity-high, immunity-medium, and immunity-low

**Fig. 2:**
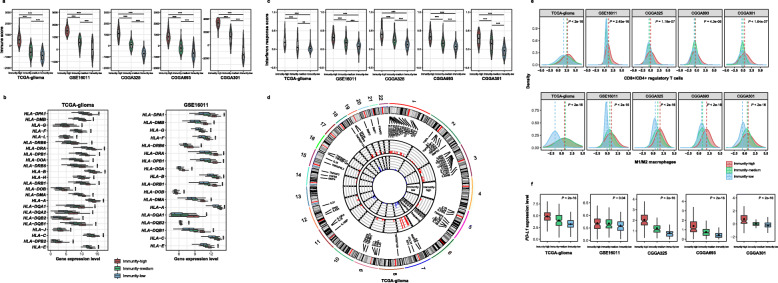
Comparisons of immune signatures between three glioma immune subtypes. Immune scores calculated by ESTIMATE [19] (**a**), the expression levels of HLA genes **(b),** interferon response scores (**c**), amplification frequencies of cytokine and cytokine receptor genes (**d**), ratios of immune-promoting/immune-inhibiting signatures (CD8+/CD4+ regulatory T cells and M1/M2 macrophages) (**e**), and *PD-L1* expression levels (**f**) were compared between immunity-high, immunity-medium, and immunity-low or between immunity-high and immunity-low subtypes of gliomas. *p < 0.05, **p < 0.01, ***p < 0.001. It also applies to the following figures

On the basis of the enrichment levels of 28 immune cell types, we hierarchically clustered gliomas in five datasets (TCGA-glioma, GSE16011, CGGA325, CGGA693, and CGGA301). We obtained three clear clusters in all five datasets: immunity-high, immunity-medium, and immunity-low (Fig. 1). The immune scores were significantly different between immunity-high, immunity-medium, and immunity-low subtypes: immunity-high > immunity-medium > immunity-low, in all five datasets (one-tailed Mann–Whitney *U* test, *P* < 0.001) (Fig. 2a). In contrast, tumor purity showed an opposite trend: immunity-high < immunity-medium < immunity-low, in all five datasets (one-tailed Mann–Whitney *U* test, *P* < 0.001) (Additional file 3: Fig. S1). Moreover, HLA genes consistently displayed markedly different expression levels in the three subtypes: immunity-high >immunity-medium > immunity-low (ANOVA test, *P* < 0.001) (Fig. 2b and Additional file 4: Fig. S2). The interferon response scores were significantly different between the three subtypes: immunity-high >immunity-medium > immunity-low (one-tailed Mann–Whitney *U* test, *P* < 0.01) (Fig. 2c). The amplification of many cytokine and cytokine receptor genes was much more frequent in immunity-high than in immunity-low in TCGA-glioma (Fig. 2d). Altogether, these data confirmed the significantly distinct tumor immune microenvironment and tumor immunity between the three subtypes.

Although the 28 immune cell types correlated with both immune-promoting and immune-inhibiting signatures, and they were consistently more highly enriched in immunity-high than in immunity-low gliomas (Fig. 1), we observed higher ratios of immune-promoting/immune-inhibiting signatures (CD8+/CD4+ regulatory T cells and M1/M2 macrophages) in immunity-high than in immunity-low gliomas (two-tailed Student’s *t* test, *P* < 0.001) (Fig. 2e). Another interesting finding was that *PD-L1* expression levels were significantly different between the three subtypes in the five datasets: immunity-high > immunity-medium > immunity-low (ANOVA test, *P* < 0.05) (Fig. 2f).

### Overlapping between the immune-specific subtyping and other subtyping methods in gliomas

We found that GBMs were mainly distributed in immunity-high and immunity-medium, while LGGs were distributed in all the three subtypes and predominated in immunity-low (Additional file 5: Fig. S3). We found that immunity-low included a higher percentage of *IDH*-mutated LGGs than immunity-high, while immunity-high included a higher percentage of *IDH*-wildtype LGGs than immunity-low (Fisher’s exact test, *P* < 0.001) (Fig. 1) in TCGA-glioma. This is consistent with previous findings that *IDH1* mutations are prevalent in LGG, which constituted a majority of immunity-low gliomas (Fig. 1). The mesenchymal and neural GBMs were mainly classified into immunity-high, and classical and proneural GBMs were mainly included in both immunity-high and immunity-low (Fig. 1). The predomination of antitumor immune responses in the mesenchymal subtype of GBM has been demonstrated in previous studies [4].

### Prediction of the immune-specific subtypes of gliomas

**Fig. 3:**
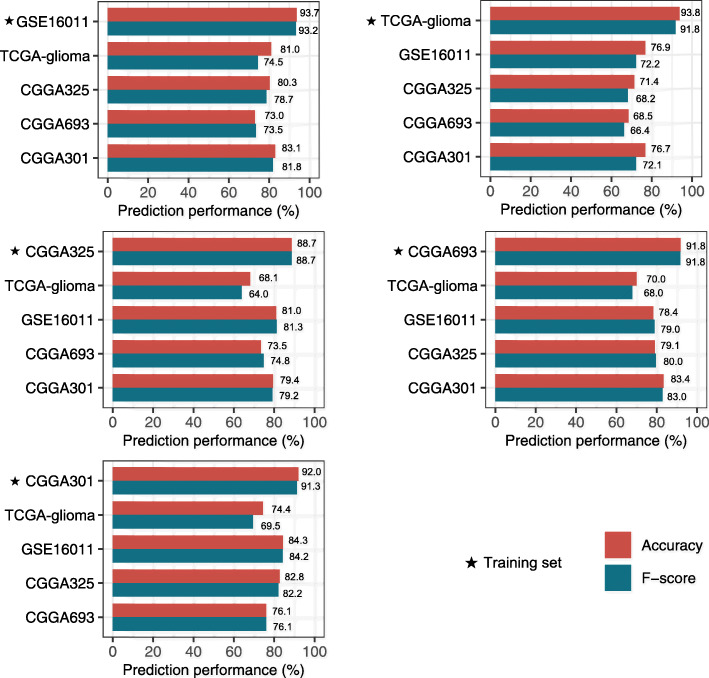
Performance in predicting the glioma immune subtypes based on the enrichment levels of 28 immune cells by the Random Forest algorithm. Each of the five datasets as the training set and the others as the test sets by turns. F-score, the weighted average of F-scores

We used GSE16011 as the training set and the other four datasets as test sets. The 10-fold cross-validation (CV) accuracy in GSE16011 was 93.7%. The prediction accuracies were 81.0%, 80.3%, 73.0%, and 83.1% in TCGA-glioma, CGGA325, CGGA693, and CGGA301, respectively (Fig. 3). The weighted F-scores in these predictions were 93.2%, 74.5%, 78.7%, 73.5%, and 81.8% for GSE16011, TCGA-glioma, CGGA325, CGGA693, and CGGA301, respectively. Furthermore, we repeated the prediction process by using TCGA-glioma, CGGA325, CGGA693, and CGGA301 as the training set, respectively, and the other datasets as test sets. We obtained similar results (Fig. 3). These data indicate that the immunological classification of gliomas is reproducible and predictable. Interestingly, the importance weights of the features (28 immune cell types) in RF varied among different training sets (Additional file 6: Fig. S4). For example, the central memory CD8 T cell had higher importance weights in TCGA-glioma and CGGA693 than in the other datasets. Several features, including effector memory CD8 T cell, myeloid-derived suppressor cell, natural killer cell, and macrophage, had high importance weights across all the datasets. In contrast, some other features, including effector memory CD4 T cell, type 17 T helper cell, CD56 dim natural killer cell, plasmacytoid dendritic cell, eosinophil, monocyte, and neutrophil, had low importance weights in all the datasets. These results suggest that some features are not important in distinguishing between the three glioma immune subtypes, and the prediction performance could improve if the unimportant features are filtered out.

### Characterizing clinical and molecular features of the immune-specific subtypes of gliomas

#### Survival prognosis

**Fig. 4:**
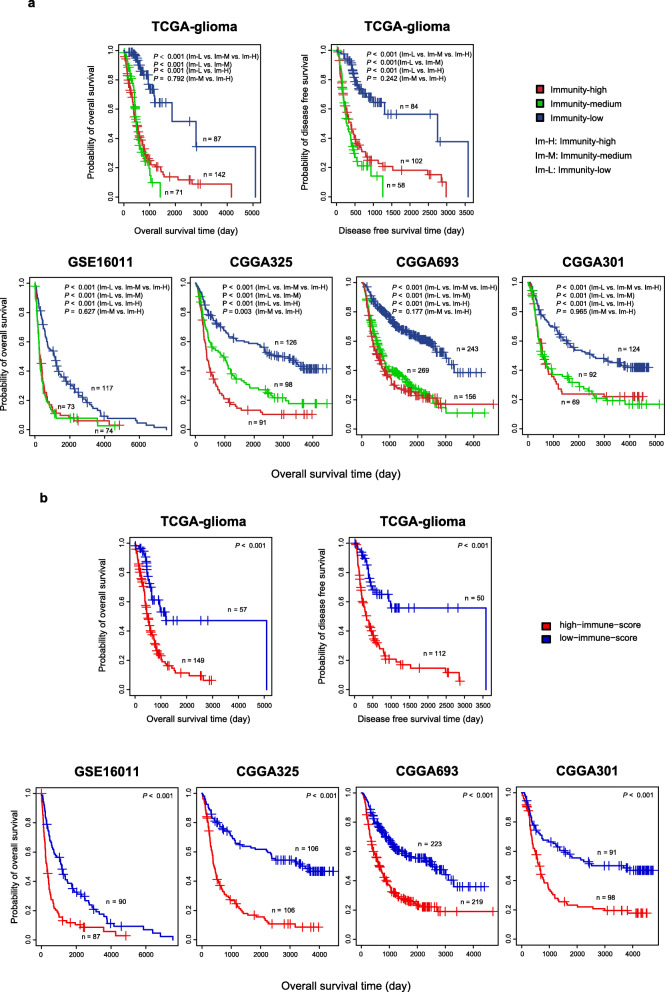
Comparisons of survival prognosis between the glioma immune subtypes and between high-immune-score (upper third) and low-immune-score (bottom third) gliomas. Kaplan–Meier curves showing that immunity-low had better overall and/or disease-free survival than immunity-medium and immunity-high (**a**) and that high-immune-score gliomas had a worse survival prognosis than low-immune-score gliomas (**b**) in the five glioma datasets. The log-rank test *p* values are shown

We found that immunity-low had better OS than immunity-medium and immunity-high in all five datasets (log-rank test, *P* < 0.001) (Fig. 4a). In contrast, the OS was not significantly different between immunity-medium and immunity-high in four datasets. Also, in the TCGA-glioma dataset, immunity-low had better DFS than immunity-medium and immunity-high (log-rank test, *P* < 0.001), and there was no significantly different DFS between immunity-medium and immunity-high (log-rank test, *P* = 0.242) (Fig. 4a). These results are inconsistent with previous studies showing that high immunity was associated with better survival in some cancers, such as triple-negative breast cancer (TNBC) [14, 27–29], indicating intertumor heterogeneity. To demonstrate that the survival difference between these subtypes is associated with their different enrichment levels of immune signatures, we compared the survival between high-immune-score (upper third) and low-immune-score (bottom third) gliomas. We found that high-immune-score gliomas had a worse survival prognosis than low-immune-score gliomas (Fig. 4b), confirming that the survival difference between the immune-specific subtypes is associated with their different immune enrichment levels. Again, the negative correlation between immune signatures and survival prognosis in gliomas is in contrast with their positive correlation shown in many other cancer types, such as TNBC [27], gastric cancer [30], and head and neck squamous cell cancer [31].

### Pathways

**Fig. 5:**
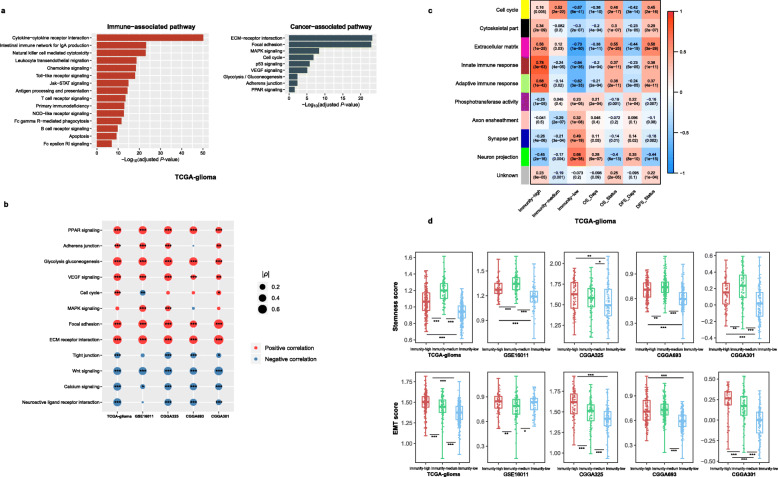
Comparisons of pathway activity, gene ontology, and tumor phenotypes between the glioma immune subtypes. **a** Immune- and cancer-associated pathways highly enriched in immunity-high versus immunity-low identified by GSEA [21]. **b** The positive correlation between cancer-associated pathways enriched in immunity-high and immune scores versus the negative correlation between the pathways enriched in immunity-low and immune scores. Spearman’s correlation test *p* values and correlation coefficients (*ρ*) are indicated. **c** Nine gene modules (gene ontology) that significantly differentiated gliomas by subtype, survival time, or survival status identified by WGCNA [22]. **d** Immunity-high has significantly higher tumor stemness and epithelial-mesenchymal transition (EMT) scores than immunity-low gliomas

We identified KEGG [20] pathways highly enriched in immunity-high and immunity-low by GSEA (Fig. 5a). As expected, the immune-associated pathways were highly enriched in immunity-high, including cytokine–cytokine receptor interactions, intestinal immune network for IgA production, natural killer cell-mediated cytotoxicity, leukocyte transendothelial migration, chemokine signaling, Toll-like receptor signaling, Jak–STAT signaling, antigen processing and presentation, B and T cell receptor signaling, NOD-like receptor signaling, Fc gamma R-mediated phagocytosis, apoptosis, Fc epsilon RI signaling, and primary immunodeficiency (Fig. 5a). This result confirmed the high immunity of immunity-high gliomas. Besides, we found many cancer-associated pathways highly enriched in immunity-high, including ECM-receptor interaction, focal adhesion, MAPK signaling, cell cycle, p53 signaling, VEGF signaling, glycolysis, adherens junction, and PPAR signaling (Fig. 5a), suggesting a positive association between these cancer-associated pathways and glioma immunity. Indeed, previous studies have revealed the positive association between cell cycle [32], p53 [30], glycolysis [33], MAPK [34], VEGF [30], and PPAR [35] and tumor immunity. In contrast, immunity-low was enriched in pathways of neuroactive ligand-receptor interaction, calcium signaling, Wnt signaling, and tight junction, suggesting an inverse association between the activities of these pathways and glioma immunity. Furthermore, the cancer-associated pathways enriched in immunity-high displayed a positive association with the immune scores, while the pathways enriched in immunity-low showed a negative correlation (Spearman’s correlation test, *P* < 0.05) (Fig. 5b).

### Gene ontology

WGCNA [22] identified nine gene modules (gene ontology) that significantly differentiated gliomas by subtype, survival time, or survival status (Fig. 5c). Consistent with previous results, the immune response was upregulated in immunity-high while it was downregulated in immunity-low (*P* < 0.001). The cell cycle was hyperactivated in immunity-high and immunity-medium while inactivated in immunity-low (*P* < 0.01). The extracellular matrix was highly enriched in immunity-high versus immunity-low (*P* < 0.01). As expected, the enrichment levels of these gene ontologies were associated with worse survival in gliomas (*P* < 0.01). In contrast, the neuron projection was highly enriched in immunity-low relative to the other subtypes (*P* < 0.01) and was associated with better survival in gliomas (*P* < 0.01).

### Tumor phenotypes

We compared several tumor phenotypes between immunity-high and immunity-low gliomas. These tumor phenotypes included tumor stemness, EMT, and grade that indicate the tumor progression. We found that immunity-high had markedly higher tumor stemness and EMT scores in five and four datasets, respectively (one-tailed Mann–Whitney *U* test, *P* < 0.01) (Fig. 5d). Immunity-high included many more high-grade tumors (grade IV) than immunity-low in TCGA-glioma and in GSE16011 (*P* < 0.01, odds ratio = 9.2). These results indicate that elevated immunity is associated with tumor progression in gliomas. This is in line with the negative correlation between tumor immunity and survival prognosis in gliomas.

### Molecular and genomic features

**Fig. 6:**
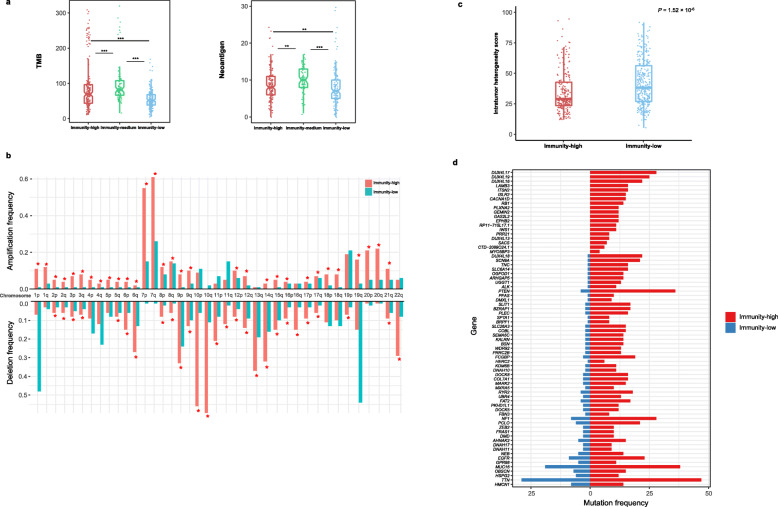
Molecular and genomic features associated with the glioma immune subtypes in TCGA-glioma. **a** Immunity-high has significantly higher tumor mutation burden (TMB) and neoantigens than immunity-low gliomas. **b** More frequent arm-level somatic copy number alterations in immunity-high versus immunity-low gliomas. The red asterisks indicate the chromosome arms in which immunity-high gliomas are more frequently amplified or deleted than immunity-low gliomas. **c** Lower intratumor heterogeneity scores in immunity-high than in immunity-low gliomas. The intratumor heterogeneity scores were evaluated by the MATH algorithm [23]. **d** Seventy-two genes more frequently mutated in immunity-high than in immunity-low gliomas

As expected, immunity-high gliomas displayed significantly higher TMB compared to immunity-low gliomas in TCGA-glioma (*P* = 7.16 × 10^−8^; median TMB, 70 versus 52) (Fig. 6a). Accordingly, the predicted tumor neoantigens [36] were more abundant in immunity-high than in immunity-low gliomas (*P* = 0.007; median *neoantigens*, 8 versus 7) (Fig. 6a). We found that arm-level SCNAs were more frequent in immunity-high than in immunity-low gliomas (*P* = 2.09 × 10^−5^, 0.001, 2.44 × 10^−5^ for amplification, deletion, and total alterations, respectively) (Fig*.* 6b). Also, focal SCNA levels were higher in immunity-high than in immunity-low gliomas (*P* = 4.14 × 10^−12^, 1.90 × 10^−12^, 4.61 × 10^−14^ for amplification, deletion, and total alterations, respectively) (Additional file 7: Fig. S5). These results indicate that immunity-high gliomas have higher levels of SCNAs than immunity-low gliomas, a finding different from that in most other cancer types [37]. Immunity-high gliomas showed lower ITH scores than immunity-low gliomas (*P* = 1.52 × 10^−6^; median ITH, 28.9 versus 38.3) (Fig. 6c). This is consistent with the fact that the ITH may lead to tumor immune evasion [38]. Interestingly, we found 72 genes more frequently mutated in immunity-high than in immunity-low gliomas (Fisher’s exact test, adjusted *P* < 0.05, odds ratio > 2) (Fig. 6d)*. * These genes included *ALK*, *DNAH10*, *11&17*, *DUX4L13*,* 16*, *17*, *18&19*, *EGFR*, *EPHB2*, *FAT2*, *KALRN*, *MAPK2*, *MUC16*, *NF1*, *PTEN*, *RB1*, and *ZEB2*, some of which were tumor suppressor genes (*NF1*, *PTEN*, and *RB1*) and oncogenes (*ALK* and *EGFR*). The mutation of the three members of DNAH genes (*DNAH10*, *11&17*) has been associated with favorable chemotherapy response [39]. In contrast, three genes (*IDH1*, *BAGE2*, and *CIC*) were more frequently mutated in Immunity-low than in Immunity-high gliomas.* IDH1* mutations are prevalent in LGG and occur early during tumorigenesis [40]. This is in accordance with our finding that Immunity-low included a high percentage of LGG samples (Fig. 1).

## Discussion

In this study, we identified immune-specific subtypes of gliomas based on the enrichment levels of 28 immune cells in the tumor environment. Our data show that gliomas can be classified into three immune subtypes: immunity-high, immunity-medium, and immunity-low. Furthermore, this immune-specific classification was stable and predictable, as shown in five different datasets. We demonstrated that immunity-low had a markedly better survival prognosis than the other subtypes, and this survival difference was associated with the different immune signature enrichment levels. These findings are contrary to those shown in many other cancer types, including TNBC [14, 27], gastric cancer [30], and head and neck squamous cell cancer [31]. It indicates the specificity of the association between tumor immunity and clinical outcomes in gliomas. A potential explanation for this is that the inflammatory tumor microenvironment promotes the progression and exacerbation of gliomas [41]. Nevertheless, immunity-medium shows no significant better survival than immunity-high, although immunity-medium has significantly lower levels of immune signatures than immunity-high (Figs. 1 and 2). The reason could be the higher TMB and stemness scores in immunity-medium versus immunity-high that worsen outcomes in immunity-medium. Likewise, we found numerous immune- and cancer-associated pathways highly enriched in immunity-high versus immunity-medium (Additional file 8: Fig. S6). Collectively, these data suggest that other factors may affect outcomes in gliomas in addition to inflammation and lymphocyte infiltration.

In the 28 immune cell types for clustering analyses, there are both immune-stimulatory (such as NK cells, activated CD8 T cells, and activated B cells) and immunosuppressive signatures (such as MDSC and regulatory T cells). We found that the enrichment levels of all these immune cells followed the same pattern: immunity-high > immunity-medium > immunity-low (Additional file 9: Fig. S7). Additionally, PD-L1 is an anti-tumor immunosuppressive molecule [42], whose expression levels also followed the pattern (Fig. 2f). Actually, the immune-stimulatory signatures are often activated in parallel with the immunosuppressive signatures [31, 33]. The ratios of immune-stimulatory/immunosuppressive signatures may determine the effect of anti-tumor immune responses. Consistent with the immune signatures, the ratios of immune-stimulatory/immunosuppressive (CD8+/CD4+ regulatory T cells and M1/M2 macrophages) were the highest in immunity-high and the lowest in immunity-low, suggesting that immunity-high and immunity-low have the strongest and weakest anti-tumor immune responses, respectively. Unfortunately, unlike many other cancer types [27, 30, 31], the elevated anti-tumor immune responses instead worsen outcomes in gliomas.

Immunity-low included a higher percentage of *IDH*-mutated and a lower percentage of *IDH*-wildtype LGGs than immunity-high, indicating that *IDH* mutations have a negative correlation with glioma immunity. This is consistent with previous study showing that *IDH* mutations were associated with low immune infiltration in gliomas [43, 44]. To exclude the potential impact of *IDH* mutations on our immunological classification of gliomas, we separated gliomas into *IDH*-wildtype and *IDH*-muted groups and compared the immune signatures between the three immune subtypes within both groups, respectively. We observed the results similar to the prior findings (Additional file 10: Fig. S8). That is, within the *IDH*-wildtype group, the enrichment levels of immune signatures followed the pattern: immunity-high > immunity-medium > immunity-low. The same pattern was also shown within the *IDH*-muted group. These data indicate that the significantly different levels of immune infiltration between the three immune subtypes of gliomas are not attributed to their significantly different mutation frequencies of *IDH*.

Besides immune signaling pathways, many cancer-associated pathways were highly enriched in immunity-high gliomas, such as MAPK signaling, cell cycle, p53 signaling, VEGF signaling, glycolysis, and PPAR signaling (Fig. 5a). Moreover, immunity-high gliomas had significantly higher tumor stemness and EMT scores and a higher percentage of high-grade tumors than immunity-low gliomas. These results indicate that immunity-high gliomas are more progressive, aggressive, and poorly prognostic than immunity-low gliomas.

Immunity-high gliomas have denser immune infiltration, active antitumor immune responses, and higher PD-L1 expression levels than immunity-low gliomas. Since both abundant immune cell infiltration [13] and high PD-L1 expression [10] are determinants of the active response to anti-PD-1/PD-L1 immunotherapy, immunity-high gliomas might have a better outcome in the immunotherapy setting. Thus, the immune-specific classification may facilitate the optimal stratification of glioma patients responsive to immunotherapy.

The immune landscape of glioma has been investigated in several recent studies [45–47]. For example, Thorsson et al. [45] identified six immune subtypes of pan-cancer and found that the immunologically quiet subtype was mostly composed of LGG, which contained the lowest level of lymphocyte infiltration. This is consistent with our result that LGGs were predominated by immunity-low. Wang et al. [46] defined three transcriptional subtypes of GBM: proneural, mesenchymal, and classical, and showed that M1 and M2 macrophages were more enriched in the mesenchymal GBMs. This is accordant with our result that the mesenchymal GBMs were mainly classified into immunity-high. Marinari et al. [47] revealed that tumor lymphocyte infiltration was an adverse prognostic factor in gliomas, consistent with our results. Overall, our unsupervised machine learning method well recaptured the immunological heterogeneity of gliomas.

## Conclusions

The antitumor immune response is an adverse prognostic factor in gliomas, a phenomenon different from that observed in other cancer types. Based on immunogenomic profiling, gliomas can be classified into three stable subtypes: immunity-high, immunity-medium, and immunity-low. Compared to immunity-low gliomas, immunity-high gliomas are more progressive, aggressive, and poorly prognostic, but could be more responsive to anti-PD-1/PD-L1 immunotherapy. The identification of immune-specific glioma subtypes has potential clinical implications for the immunotherapy of gliomas.

## Supplementary Information

**Additional file 1: Table S1.** A summary of five glioma datasets used in this study.

**Additional file 2: Table S2.** The gene sets representing immune cells or signatures, pathways, and biological processes.

**Additional file 3: Fig. S1.** Comparison of tumor purity between three glioma immune subtypes.

**Additional file 4: Fig. S2.** Comparison of the expression levels of HLA genes between three glioma immune subtypes.

**Additional file 5: Fig. S3.** Overlapping between the immune-specific subtyping and other subtyping methods in gliomas.

**Additional file 6: Fig. S4.** Importance weights of the 28 features (immune cell types) in the training set for the Random Forest classifier.

**Additional file 7: Fig. S5.** More frequent focal somatic copy number alterations in Immunity-high versus Immunity-low gliomas.

**Additional file 8: Fig. S6.** Immune- and cancer-associated pathways highly enriched in Immunity-high versus Immunity-medium gliomas.

**Additional file 9: Fig. S7.** Comparison of the enrichment levels of 28 immune cells between three glioma immune subtypes.

**Additional file 10: Fig. S8.** Comparison of the enrichment levels of immune signatures between three glioma immune subtypes within *IDH*-wildtype and *IDH*-muted groups.

## Data Availability

The TCGA-glioma (GBM and LGG) datasets were downloaded from the TCGA data portal (https://portal.gdc.cancer.gov/). GSE16011 were downloaded from the NCBI gene expression omnibus (https://www.ncbi.nlm.nih.gov/geo/). CGGA301, CGGA325, and CGGA693 were downloaded from the Chinese Glioma Genome Atlas (http://www.cgga.org.cn/). A summary of these datasets is presented in Additional file 1: Table S1.

## Abbreviations

LGG: Lower-grade glioma
GBM: Glioblastoma
TCGA: The Cancer Genome Atlas
TIM: Tumor immune microenvironment
HLA: Human leukocyte antigen
EMT: Epithelial-mesenchymal transition
TMB: Tumor mutation burden
SCNA: Somatic copy number alteration
ssGSEA: Single-sample gene set enrichment analysis
NK: Natural killer
MDSC: Myeloid-derived suppressor cell
OS: Overall survival
DFS: Disease-free survival
WGCNA: Weighted gene co-expression network analysis
GSEA: Gene set enrichment analysis
ITH: Intratumor heterogeneity
RF: Random forest
CV: Cross validation
TNBC: Triple-negative breast cancer
