# Supplementary figures and images for: Immunological classification of gliomas based on immunogenomic profiling
Source: J Neuroinflammation. 2020 Nov 27;17:360. doi: 10.1186/s12974-020-02030-w (PMC7694942; doi:10.1186/s12974-020-02030-w)

Figure S1

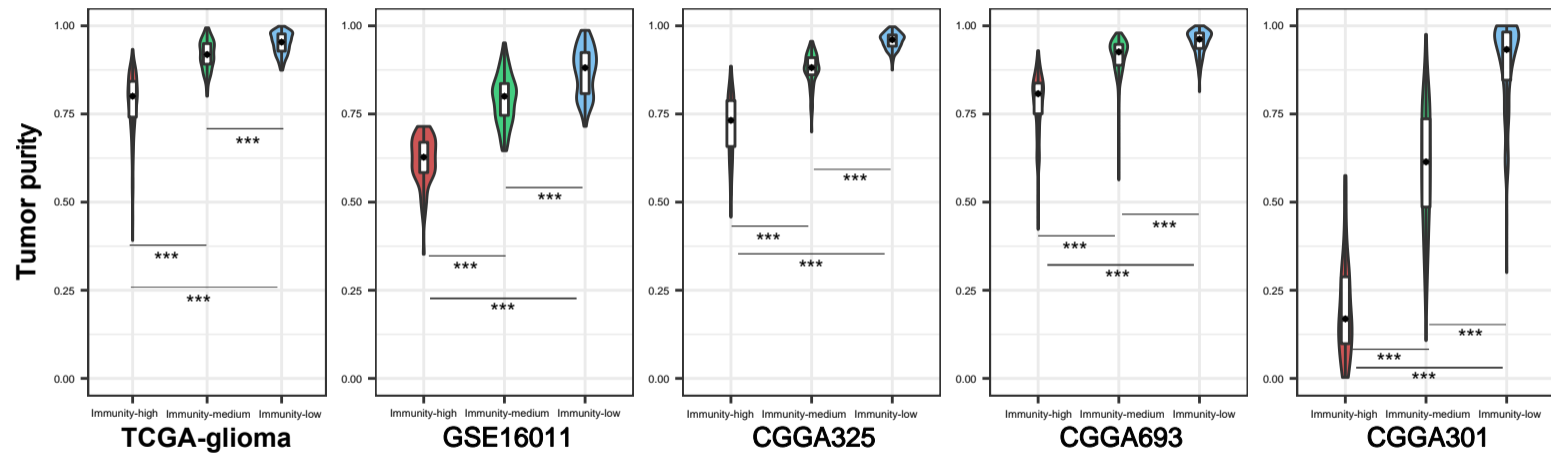

Supplement: Supplementary file 3 — Additional file 3: Fig. S1. Comparison of tumor purity between three glioma immune subtypes. [file 12974_2020_2030_MOESM3_ESM.pdf]

Figure S2

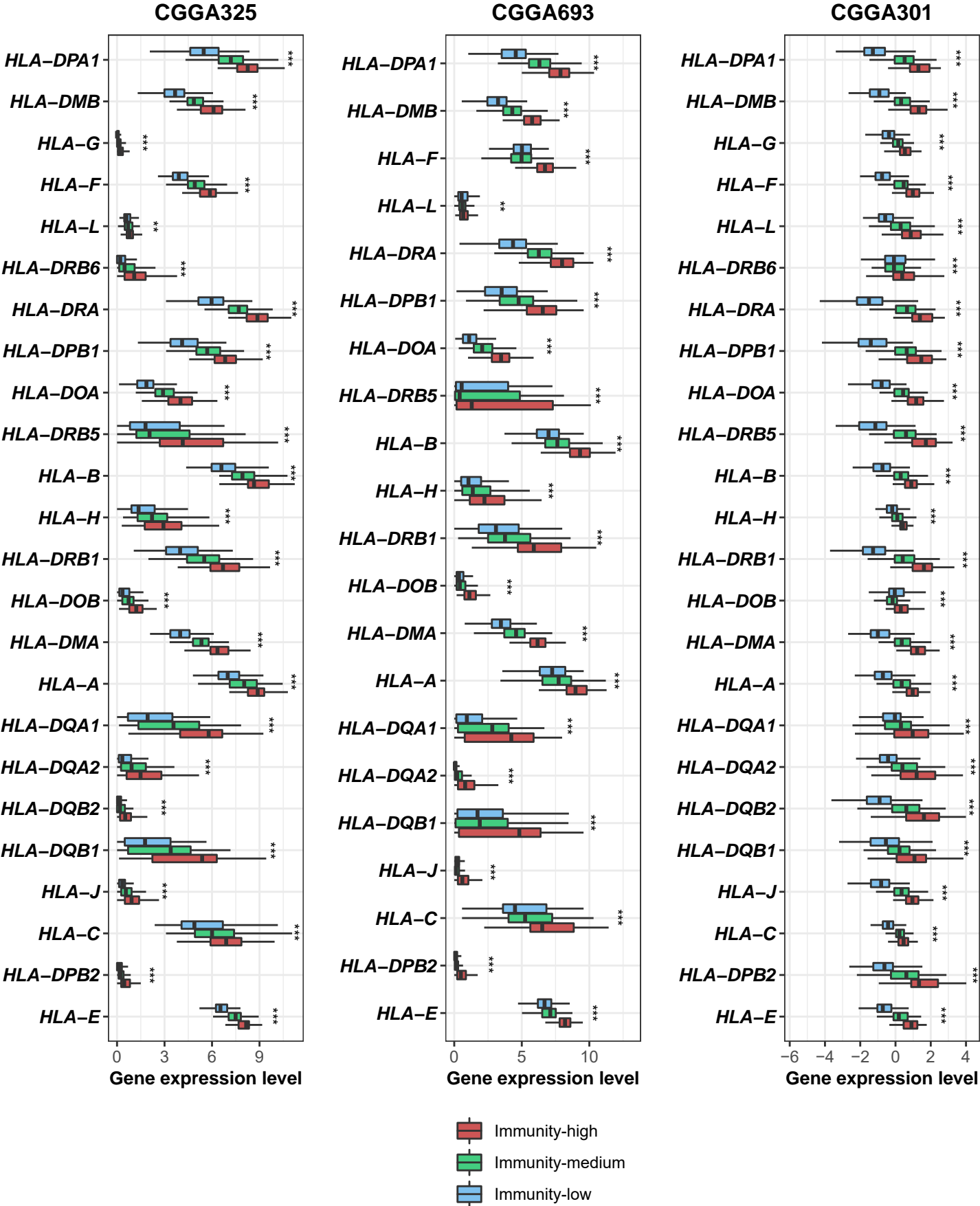

Supplement: Supplementary file 4 — Additional file 4: Fig. S2. Comparison of the expression levels of HLA genes between three glioma immune subtypes. [file 12974_2020_2030_MOESM4_ESM.pdf]

Figure S3

TCGA-glioma

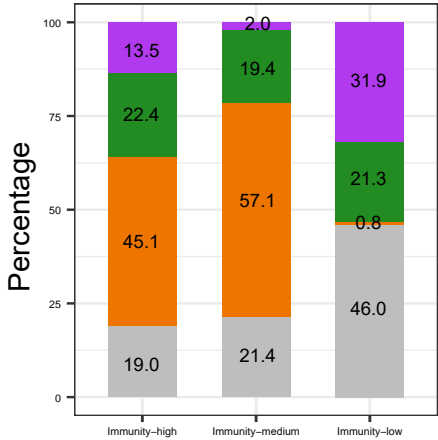

GSE16011

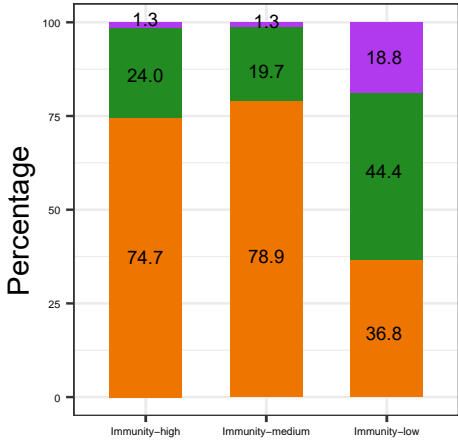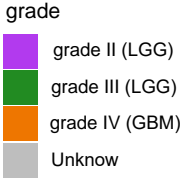

CGGA325

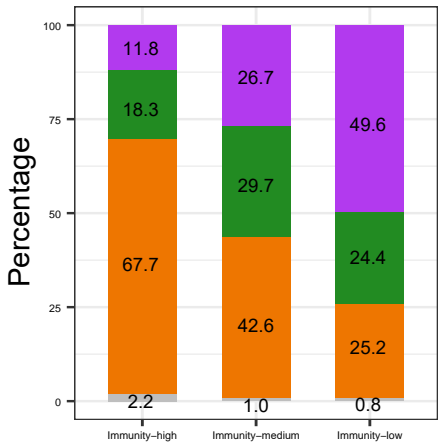

CGGA693

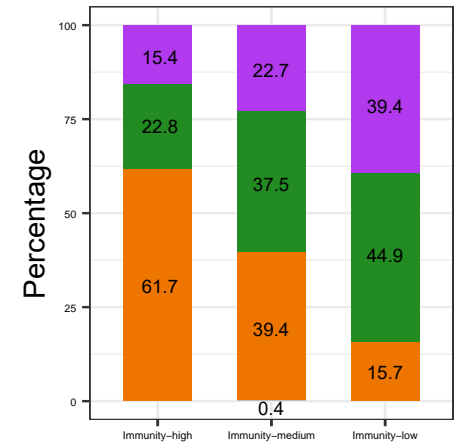

CGGA301

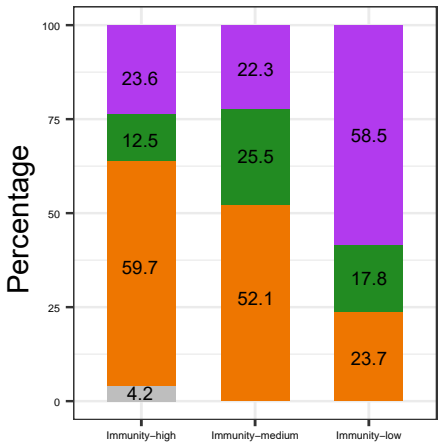

TCGA-glioma

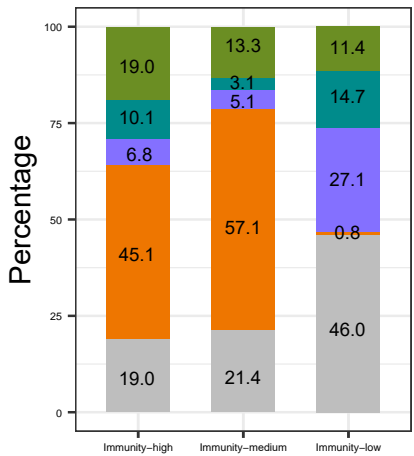

GSE16011

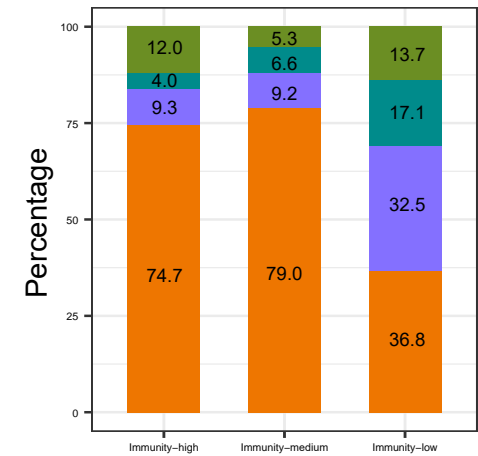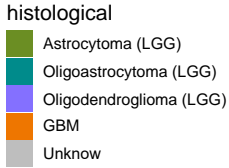

CGGA325

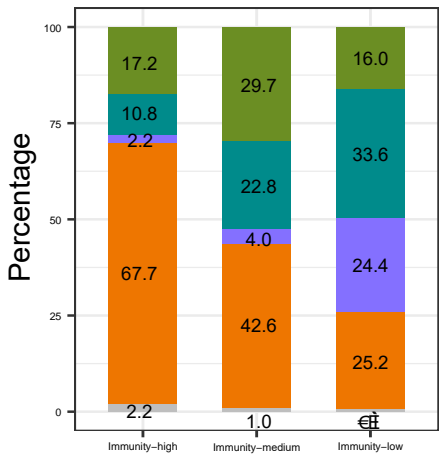

CGGA693

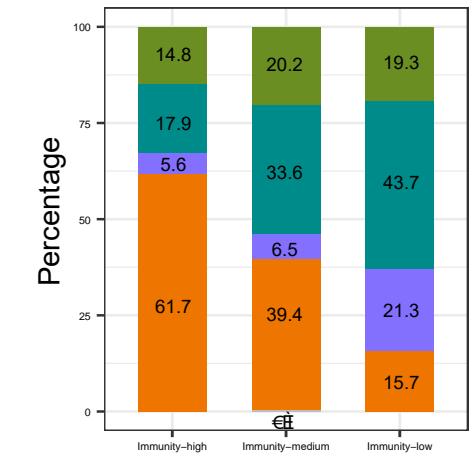

CGGA301

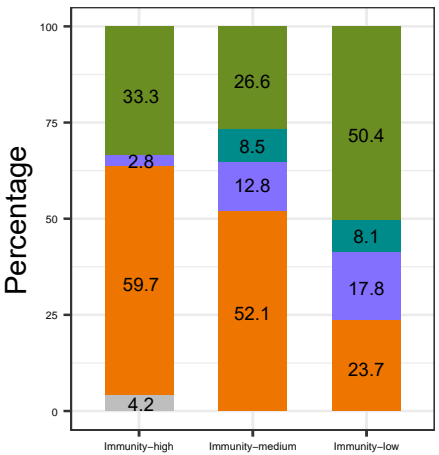

Supplement: Supplementary file 5 — Additional file 5: Fig. S3. Overlapping between the immune-specific subtyping and other subtyping methods in gliomas. [file 12974_2020_2030_MOESM5_ESM.pdf]

Figure S4

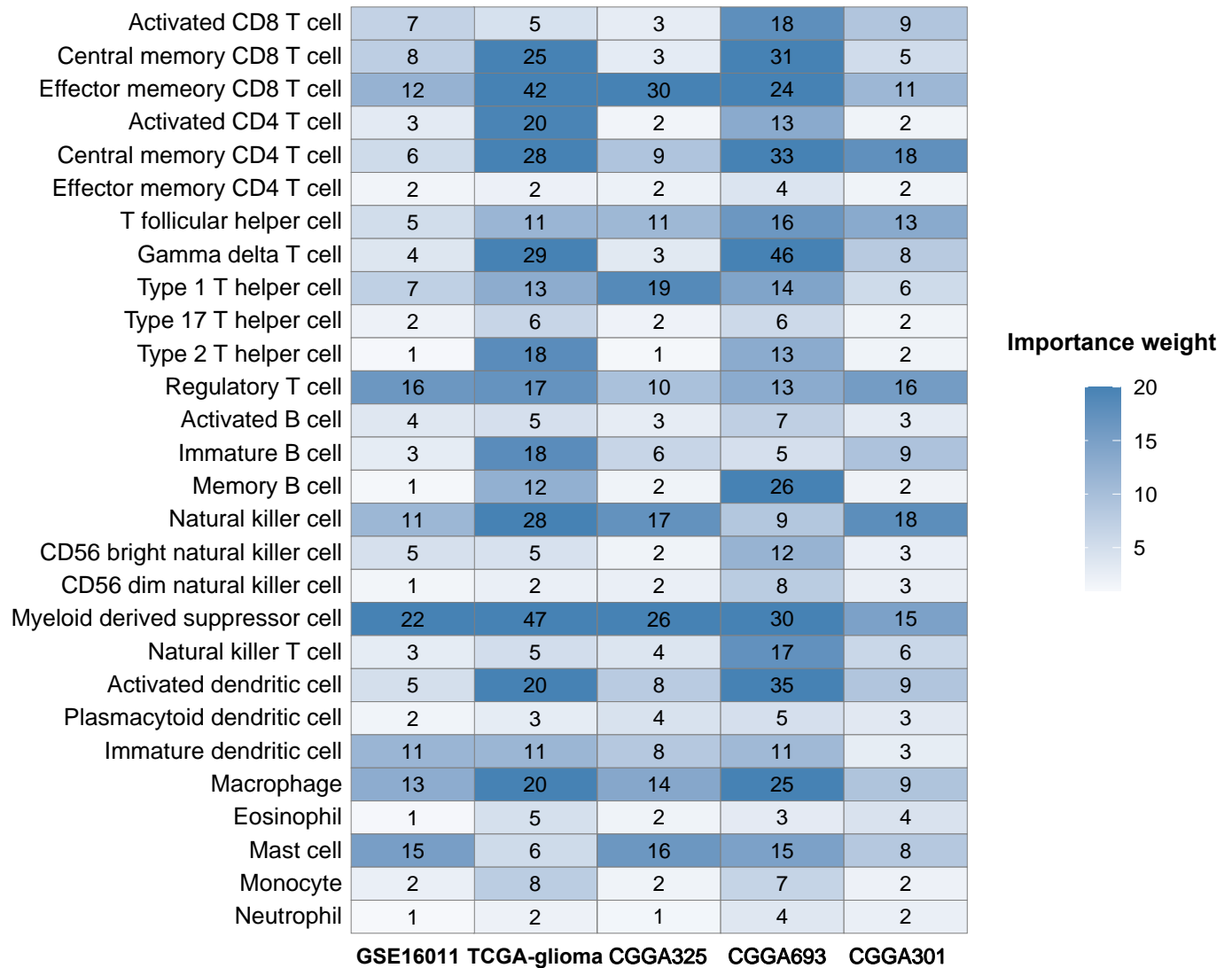

Supplement: Supplementary file 6 — Additional file 6: Fig. S4. Importance weights of the 28 features (immune cell types) in the training set for the Random Forest classifier. [file 12974_2020_2030_MOESM6_ESM.pdf]

Figure S5

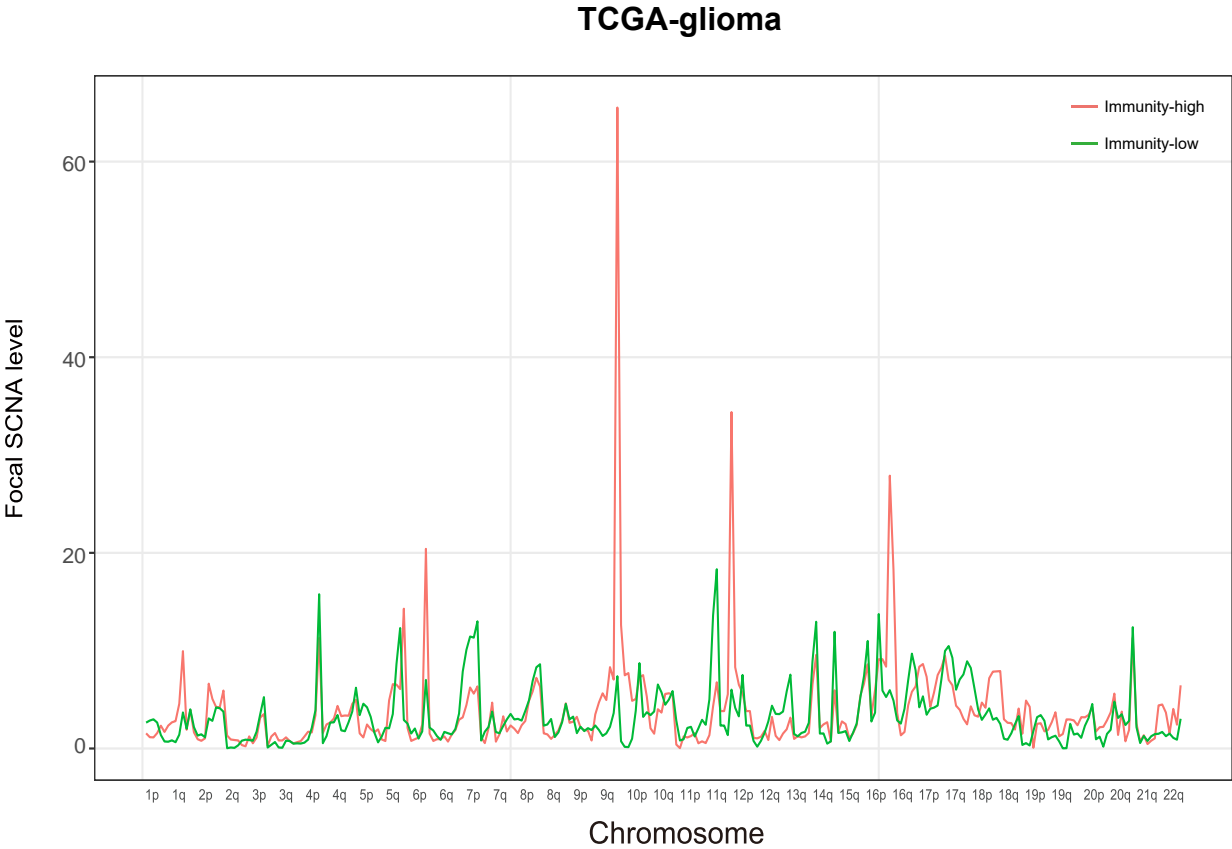

Supplement: Supplementary file 7 — Additional file 7: Fig. S5. More frequent focal somatic copy number alterations in Immunity-high versus Immunity-low gliomas. [file 12974_2020_2030_MOESM7_ESM.pdf]

Figure S6

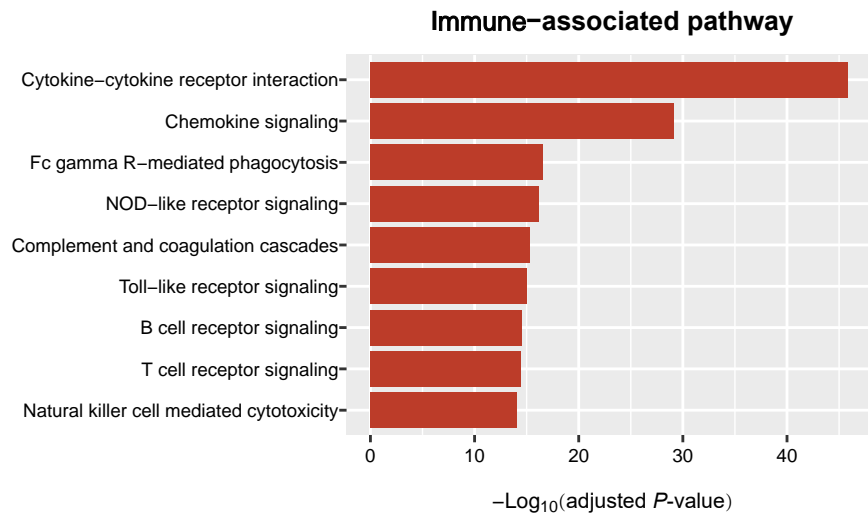

**TCGA-glioma**

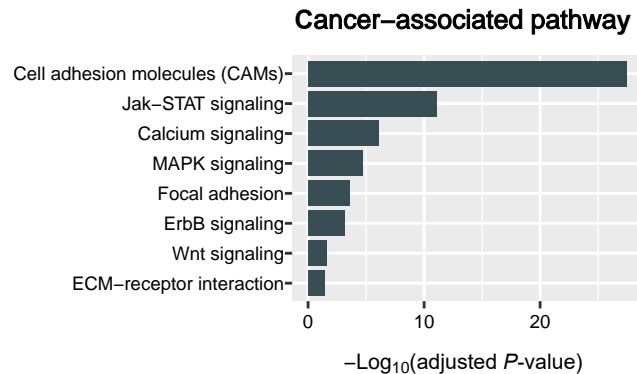

Supplement: Supplementary file 8 — Additional file 8: Fig. S6. Immune- and cancer-associated pathways highly enriched in Immunity-high versus Immunity-medium gliomas. [file 12974_2020_2030_MOESM8_ESM.pdf]

Figure S7

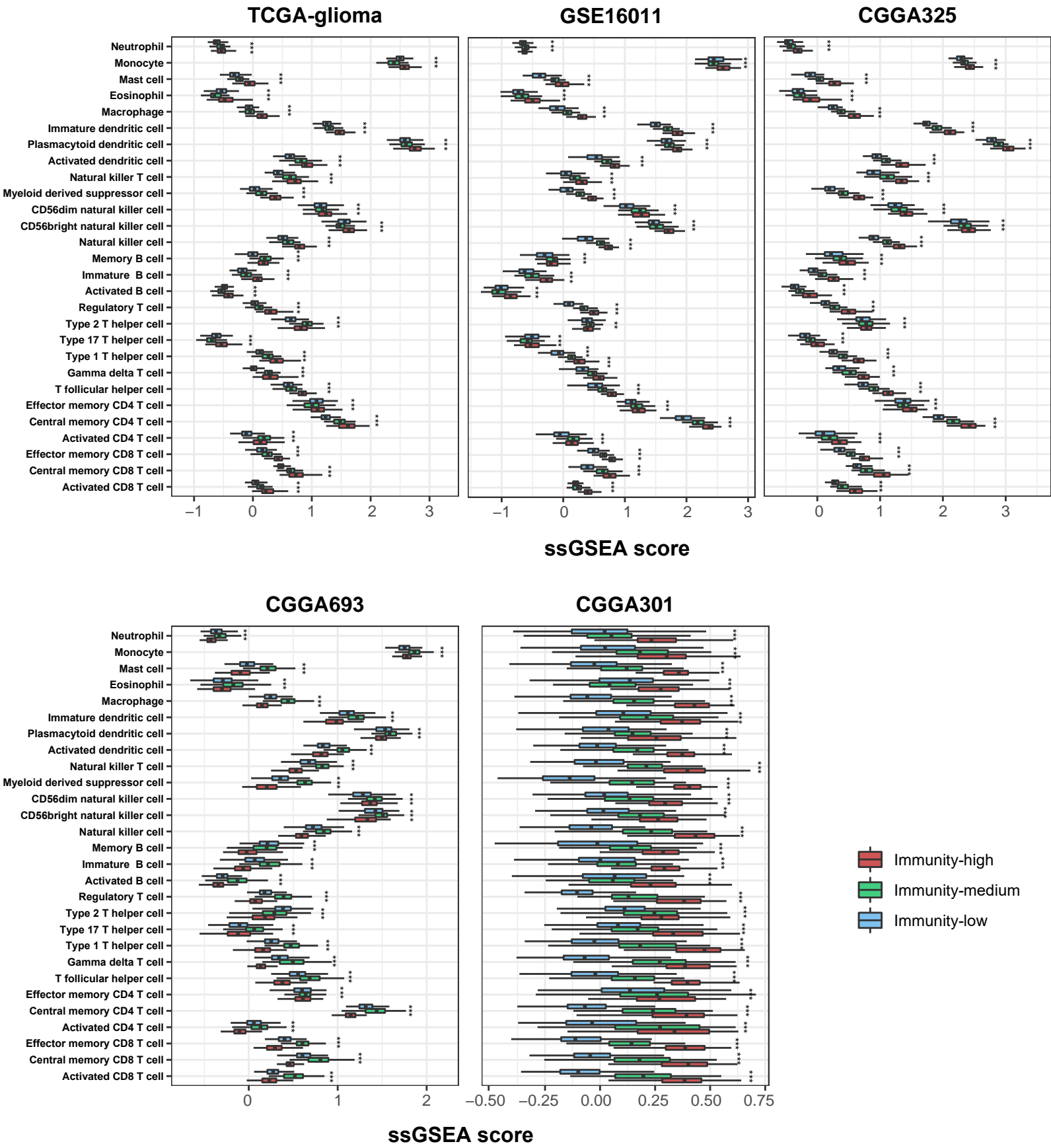

Supplement: Supplementary file 9 — Additional file 9: Fig. S7. Comparison of the enrichment levels of 28 immune cells between three glioma immune subtypes. [file 12974_2020_2030_MOESM9_ESM.pdf]

Figure S8

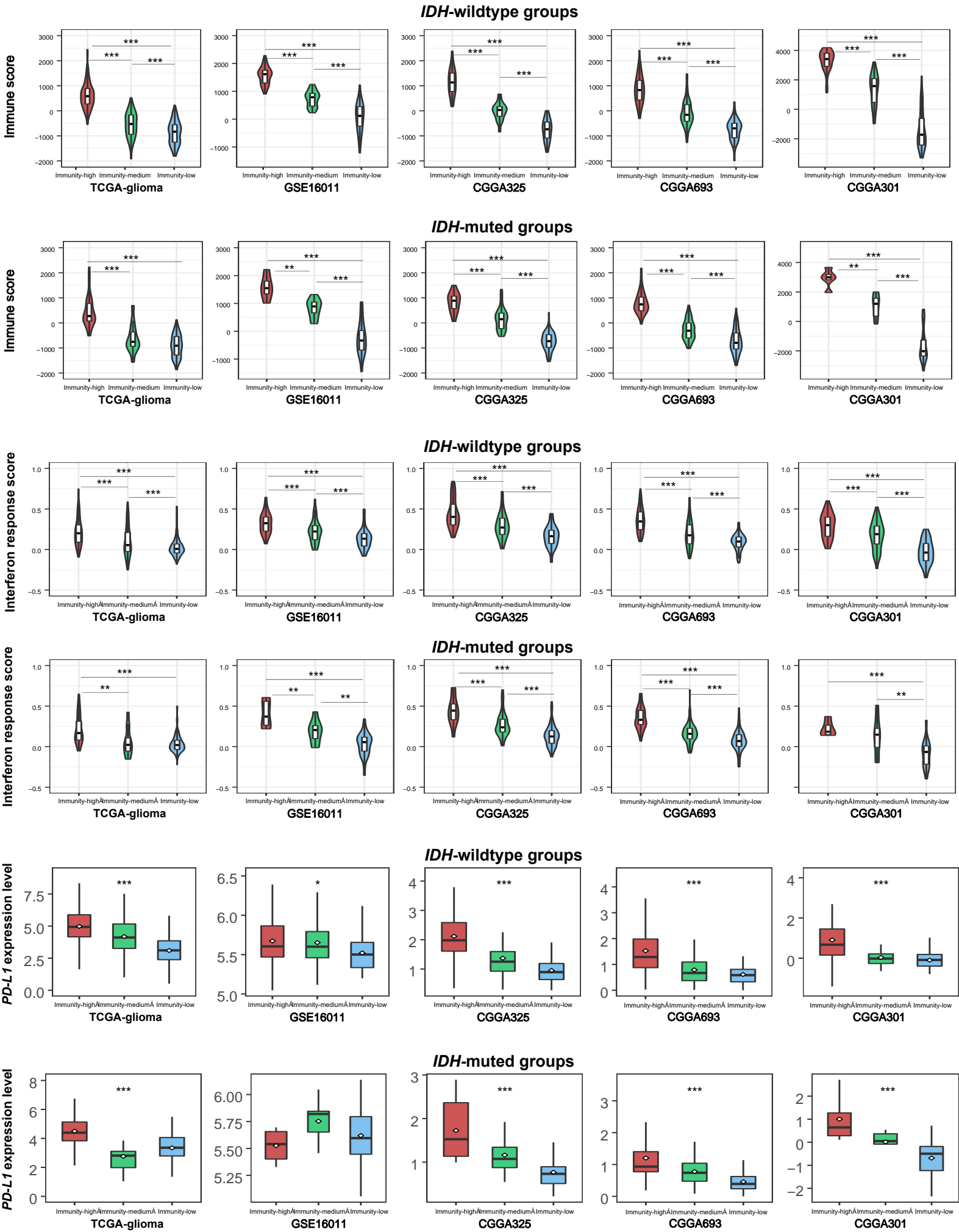

Supplement: Supplementary file 10 — Additional file 10: Fig. S8. Comparison of the enrichment levels of immune signatures between three glioma immune subtypes within IDH-wildtype and IDH-muted groups. [file 12974_2020_2030_MOESM10_ESM.pdf]
